# Supplementary material for: High-Spatial-Resolution Benchtop X-ray Fluorescence Imaging through Bragg-Diffraction-Based Focusing with Bent Mosaic Graphite Crystals: A Simulation Study
Source: Int J Mol Sci. 2024 Apr 26;25(9):4733. doi: 10.3390/ijms25094733 (PMC11083219; doi:10.3390/ijms25094733)
Supplement: Supplementary file 1 [file ijms-25-04733-s001.zip › ijms-2940389-supplementary.pdf]

Supplementary Materials: High-Spatial-Resolution Benchtop X-ray Fluorescence Imaging through Bragg-Diffraction-Based Focusing with Bent Mosaic Graphite Crystals: A Simulation Study

Kunal Kumar, Melanie Fachet † and Christoph Hoeschen

†Correspondence: melanie.fachet@ovgu.de

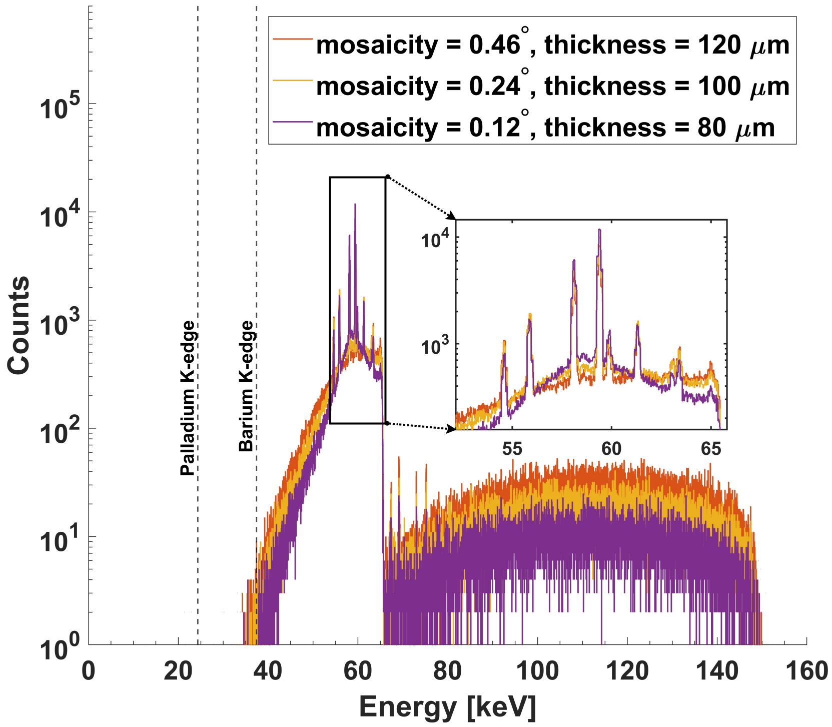

Figure S1. Comparison of focused X-ray spectra at $f = 320\,\text{mm}$ for crystals with mosaic spreads of $m = 0.12°$, $m = 0.24°$, and $m = 0.46°$ using ray-tracing simulations on *mmpxrt* [1].

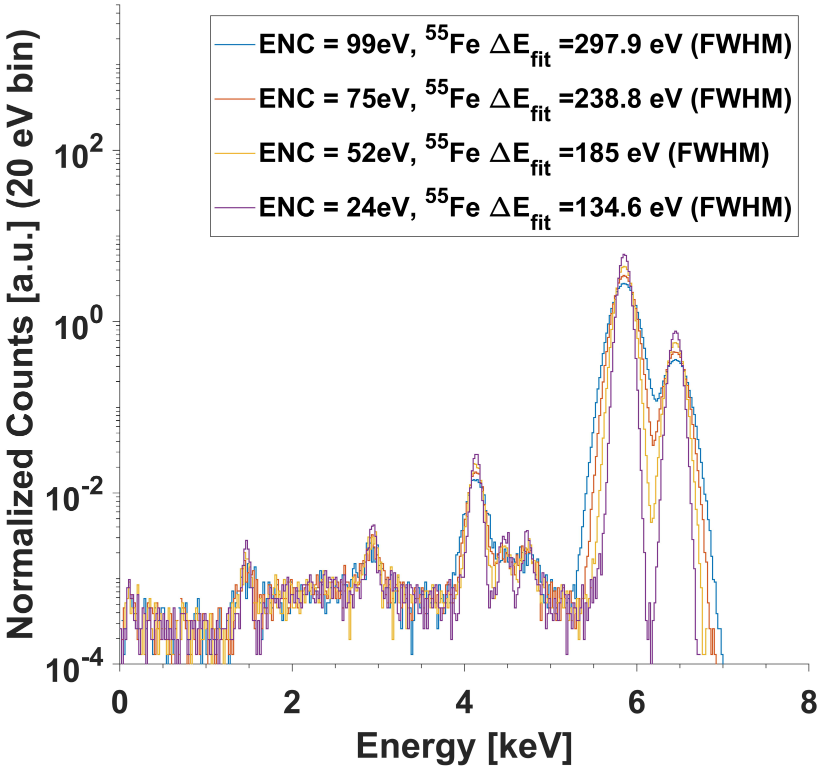

Figure S2. Simulated spectra of ^{55}Fe radioactive source with 1 mm thick, 70 mm^2 area - collimated to 40 mm^2, silicon drift detectors (XR-100FastSDD, Amptek Inc., MA, USA [2]). The energy resolution (ΔE_{fit} eV FWHM) estimated for 5.9 keV K_α X-rays (photopeak from decay to ^{55}Mn) is compared across various levels of simulated electronic noise (equivalent noise charge, ENC). The estimated energy resolution and spectra shape is in good agreement with the typical resolution values ($\sim$ 124 eV to $\sim$ 148 eV, depending on the output count rate) and the measured spectra reported in the reference [2,3]. For XFI simulations, a conservative value of ENC = 75 eV is assumed. Considering the unavailability of measured raw data (X-ray spectra) from Amptek and incomplete information regarding the experimental conditions, it is acknowledged that there may be aspects requiring further evaluation.

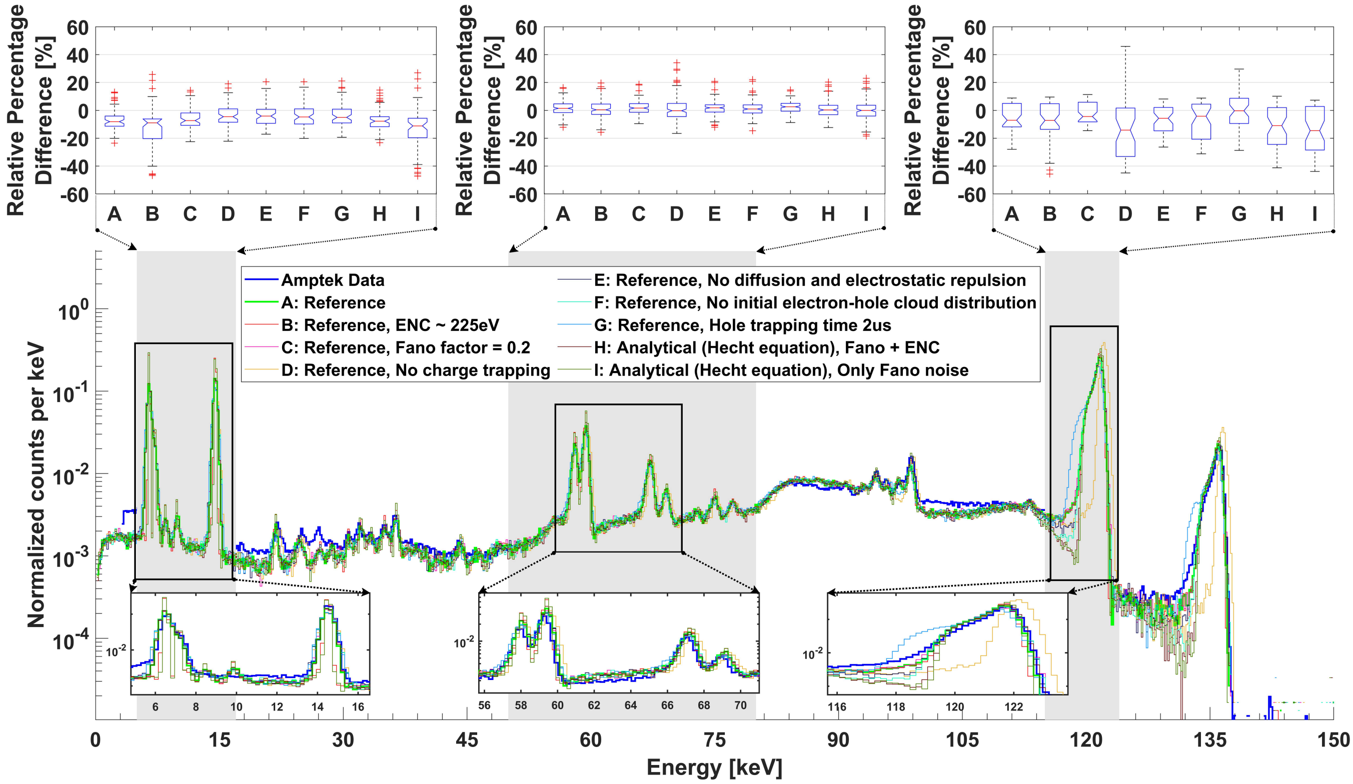

Figure S3. Simulated spectra of ^{57}Co radioactive source with 1 mm thick, 25 mm^2 area, CdTe detector (XR-100CdTe, Amptek Inc., MA, USA [6]). A sensitivity analysis is conducted to assess the influence of individual components (i.e., detector physical properties and electronics) in the detector model on the overall detector response. The relative percentage differences from the experimental data (provided by Amptek) are presented for the different energy ranges, indicated by shaded grey areas. The Amptek data is compared with the implemented high-fidelity model (labeled A: reference), followed by changes to this model. Model A is employed for the XFI simulations with CdTe detector arrangement. Model B incorporates changes in ENC from 358 eV to 225 eV. Model C involves changes in the Fano factor from 0.1 to 0.2. In model D, charge trapping is excluded. In model E, charge diffusion and electrostatic repulsion are excluded. In model F, the formation of the initial electron-hole cloud is excluded. In model G, the hole trapping time is adjusted from 3 μs to 2 μs. Model H and I are analytical models derived from the Hecht equation (see Equation 1). Here, model H incorporates energy blurring from Fano broadening and electronic noise, while model I excludes Gaussian broadening from electronic noise. Overall, model A is in good agreement with the Amptek data.

For analytical models, the Hecht equation is used to model the efficiency of charge collection $\eta(z)$, i.e., the total induced charge per created charge, as a function of depth z [4,5]:

$$\eta(z) = \left(\frac{\lambda_e}{T_{det}}\right)\left(1 - \exp\left[\frac{z - T_{det}}{\lambda_e}\right]\right) + \left(\frac{\lambda_h}{T_{det}}\right)\left(1 - \exp\left[-\frac{z}{\lambda_h}\right]\right), \tag{S1}$$

where $T_{det} = 1$ mm is the detector thickness and λ_e, λ_h are the trapping lengths of electrons and holes, respectively. The trapping lengths are given as $\lambda_e = \mu_e \tau_e v / T_{det}$, where mobility is $\mu_e = 1100\,\mathrm{cm}^2/(\mathrm{V}\cdot\mathrm{s})$, $\mu_h = 100\,\mathrm{cm}^2/(\mathrm{V}\cdot\mathrm{s})$, trapping time is $\tau_e = \tau_h = 3\,\mu$s, and bias is $v = 800$ V.

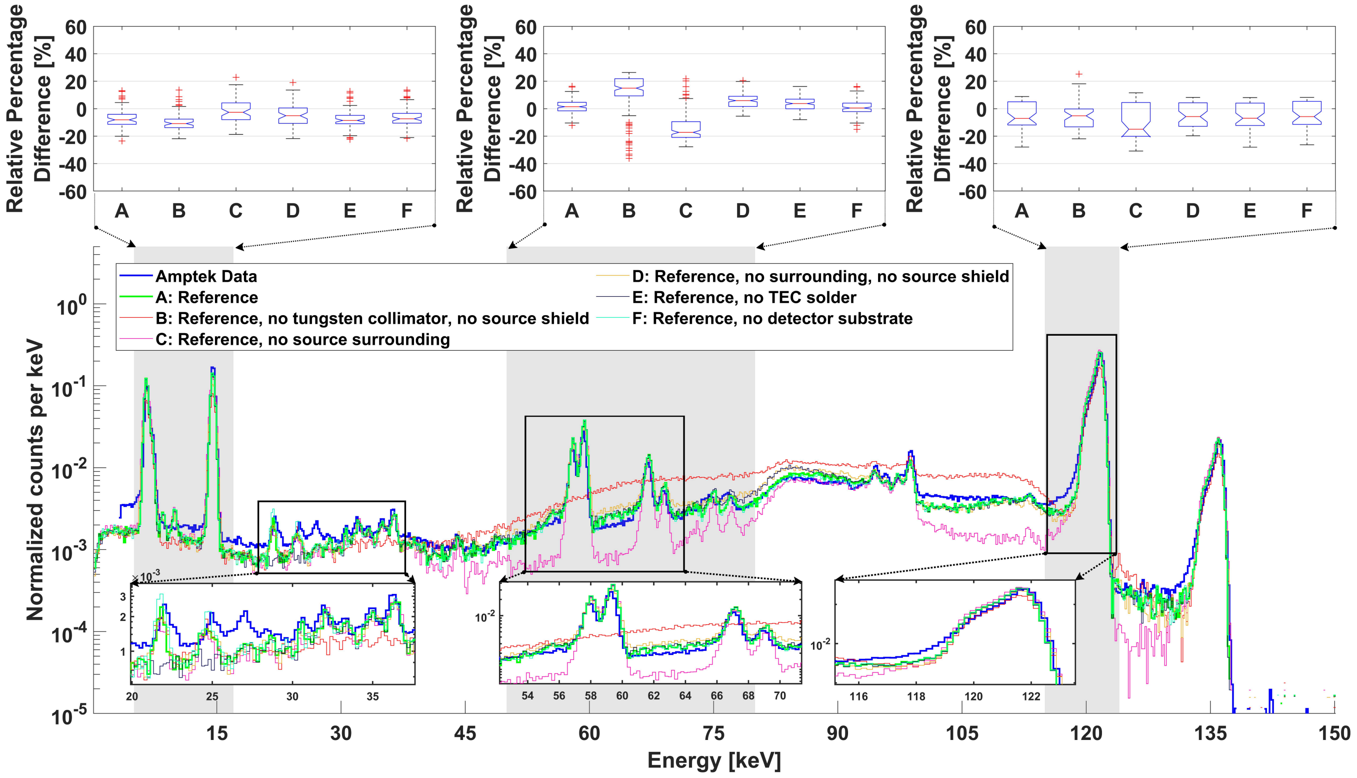

Figure S4. Influence of geometric effects on the simulated spectra of ^{57}Co radioactive source using CdTe. Here, the sensitivity analysis evaluates the impact of simulated geometric components (i.e., detector components, radioactive source geometry, and the surrounding environment) on the overall detector response. Relative percentage differences from Amptek's data are shown for different energy ranges (shaded grey areas). Only cases with the most substantial deviations from the high-fidelity model are presented. The Amptek data is compared with the implemented high-fidelity model (labeled A: reference), followed by changes in the geometric components. In model B, the radioactive capsule implementation excludes the tungsten collimator and the shielding elements present in model A. In model C, the surrounding geometry of the source is excluded. In model D, all surrounding components within the environment, including the shielding elements of the source, are excluded. In model E, the solder elements, primarily composed of tin (Sn), are excluded from the detector's thermoelectric cooler (TEC). In model F, the detector's substrate, primarily composed of alumina (Al_2O_3), is excluded. Overall, model A is in good agreement with the Amptek data.

References

1. Šmíd, M.; Pan, X.; Falk, K. X-ray spectrometer simulation code with a detailed support of mosaic crystals. *Comput. Phys. Commun.* **2021**, *262*, 107811.
2. Amptek Silicon Drift Detector XR-100FastSDD. Available online: https://www.amptek.com/-/media/ametekamptek/documents/resources/products/user-manuals/xr100-1mm-fastsdd-user-manual-b4.pdf?la=en&revision=24e8eb09-6164-48ba-8336-e572f84bf5c1 (accessed on 15 December 2023).
3. Application Notes: Amptek Silicon Drift Detector XR-100FastSDD. Available online: https://www.amptek.com/-/media/ametekamptek/documents/resources/application-notes/high-sensitivity-detectors-for-xrf.pdf?la=en&revision=9d04dd37-c2ea-4f89-ad58-55579a8574b1 (accessed on 15 December 2023).
4. Application Note ANCZT-2 Rev. 3. Charge Trapping in XR-100T-CdTe and -CZT Detectors. Available online: https://www.amptek.com/-/media/ametekamptek/documents/resources/application-notes/charge-trapping-in-cdte-and-czt.pdf?la=en&revision=6aab2ea7-e5be-43bd-97c5-bc2407f1503a. (accessed on 15 December 2023).
5. Redus, R.H.; Pantazis, J.A.; Pantazis, T.J.; Huber, A.C.; Cross, B.J. Characterization of CdTe detectors for quantitative X-ray spectroscopy. *IEEE Trans. Nucl. Sci.* **2009**, *56*, 2524–2532.

6. Amptek Cadmium Telluride Detector XR-100CdTe. 2017 Available online: https://www.amptek.com/-/media/ametekamptek/documents/resources/products/user-manuals/xr100-cdte-user-manual-b2.pdf?la=en&revision=c7533c8d-5c70-4172-a1e0-3582edb70d5e (accessed on 15 December 2023).

Disclaimer/Publisher's Note: The statements, opinions and data contained in all publications are solely those of the individual author(s) and contributor(s) and not of MDPI and/or the editor(s). MDPI and/or the editor(s) disclaim responsibility for any injury to people or property resulting from any ideas, methods, instructions or products referred to in the content.
